# Moderate tea consumption and dementia-related neuroimaging markers

**DOI:** 10.3389/fneur.2025.1634621

**Published:** 2025-11-11

**Authors:** Ting Jin, Dongwei Lu, Jiyu Chen, Wenxuan Zhao, Yanfang Zhang, Qing Zhang, Xujun Ye, Juanjuan Qin

**Affiliations:** 1 Department of Geriatrics, Zhongnan Hospital of Wuhan University, Wuhan University, Wuhan, China; 2 Department of General Practice, Zhongnan Hospital of Wuhan University, Wuhan University, Wuhan, China; 3 Department of Interventional Ultrasound, Tianyou Hospital Affiliated to Wuhan University of Science and Technology, Wuhan, China; 4 Department of Neuropsychology, Zhongnan Hospital of Wuhan University, Wuhan, China; 5 Center for Healthy Aging, Wuhan University School of Nursing, Wuhan, China

**Keywords:** tea consumption, neuroimaging, dementia, cognition, UK Biobank

## Abstract

**Introduction:**

Tea consumption may be associated with a reduced risk of dementia. However, the association between tea consumption and dementia-related neuroimaging markers remains unclear.

**Methods:**

We analyzed 438,078 dementia-free participants from the UK Biobank at baseline, including 38,584 with complete brain imaging data. Linear regression models assessed brain imaging, Cox proportional hazards models evaluated dementia risk, and logistic regression analyzed cognitive decline. All analyses were adjusted for covariates and stratified by sex and age.

**Results:**

Moderate tea consumption was positively associated with regional brain volumes, including gray matter volume in the anterior parahippocampal gyrus, cuneal cortex, and the frontal lobe. Daily consumption of 6-7 cups of tea was significantly negatively associated with volumes of white matter hyperintensity (WMH) and the anterior cingulate cortex (ACC). Additionally, a significant association with the anterior parahippocampal gyrus was observed only in males. We found tea consumption showed a non-linear (*p* for non-linear < 0.0001) association with dementia. The lowest risk of incident dementia at a daily consumption level of 4–5 cups of tea (fully adjusted HR 0.804, 95% CI 0.752, 0.861) compared to non-consumption, consistent with the neuroimaging findings. No association was observed with cognitive decline.

**Conclusion:**

Moderate tea consumption was associated with volumes in several brain regions and reduced risk of dementia. This study comprehensively demonstrates the consistent associations of moderate tea consumption with dementia risk and brain health, highlighting the potential benefits of moderate tea consumption in preventing dementia.

## Background

With the increasing prevalence of an aging population, dementia has emerged as a critical global public health challenge. According to the World Health Organization, approximately 55 million individuals are currently living with dementia worldwide, a figure anticipated to rise to 139 million by 2050 (1). This growing prevalence places a heavy medical and economic burden on societies. There remains no effective cure for dementia. Therefore, identifying effective prevention and management strategies is of utmost importance.

Tea is the most popular and widely consumed beverage globally. It contains abundant catechin polyphenols, caffeine, and flavonoid compounds, which have neuroprotective effects, including oxidative stress, anti-inflammatory, immune-regulation, and inhibition of β-amyloid aggregation (2, 3). Tea consumption has been found to be associated with the prevention of various diseases, such as diabetes, cardiovascular diseases, and neurodegenerative diseases (4–6). In recent years, a growing number of studies have suggested that tea drinking may slow brain aging and improve cognitive dysfunction (7, 8). However, some studies have found no association between tea consumption and dementia risk (9). This inconsistency may be related to the follow-up period and differences in the population.

The pathological process of neurodegenerative diseases may have a preclinical stage of more than 10 years (10), and alterations in the brain can be detected through magnetic resonance imaging. Previous studies have suggested a potential influence of tea consumption on the brain, positing that polyphenols may activate neuronal cells to mitigate brain atrophy (11). However, the results are inconsistent, likely due to the limitations of sample size and estimation method. One of the earliest functional magnetic resonance imaging studies found that green tea significantly increased activation in the dorsolateral prefrontal cortex, which in turn regulates the brain's working memory (12). Another study demonstrated that green tea acts on working memory to improve performance on cognitive tasks by increasing connectivity from the right parietal to the middle frontal gyrus (13). A recent Japanese prospective cohort study with 2 years of follow-up suggested that higher green tea intake was associated with a reduction in annual hippocampal atrophy, which was most pronounced in older adults and women (14). However, the extent to which tea consumption may mitigate the risk of dementia through modifications in brain morphology remains unclear. Investigating the relationship between tea intake and brain health could shed light on its potential role in preventing dementia.

In this study, we utilized data from the large-scale UK Biobank study—a longitudinal cohort with a median follow-up of 14 years—to investigate the association between tea consumption and the risk of incident dementia among middle-aged and older adults. Additionally, we examined the association between tea consumption and brain neuroimaging biomarkers, as well as cognitive decline.

## Materials and methods

### Study design and population

This study is based on the UK Biobank (UKB), which is an ongoing large-scale prospective study. Baseline data were collected from 502,359 participants aged 37–73 years between 2006 and 2010 from 22 assessment centers in England, Wales, and Scotland, providing a wide range of information through questionnaires, interviews, health records, physical measurements, and blood samples (15). Since recruitment, participants were followed for clinical outcomes, including dementia, through hospital hospitalization records, death certificates, and primary care records. Brain magnetic resonance imaging (MRI) scan began in 2014 with a subsample of 100,000 participants (16). The UK Biobank study has been approved by the National Health and Social Care Information Management Board and the NHS North West Multicenter Research Ethics Committee (11/NW/03 820), and all participants signed written consent forms.

**Figure 1:**
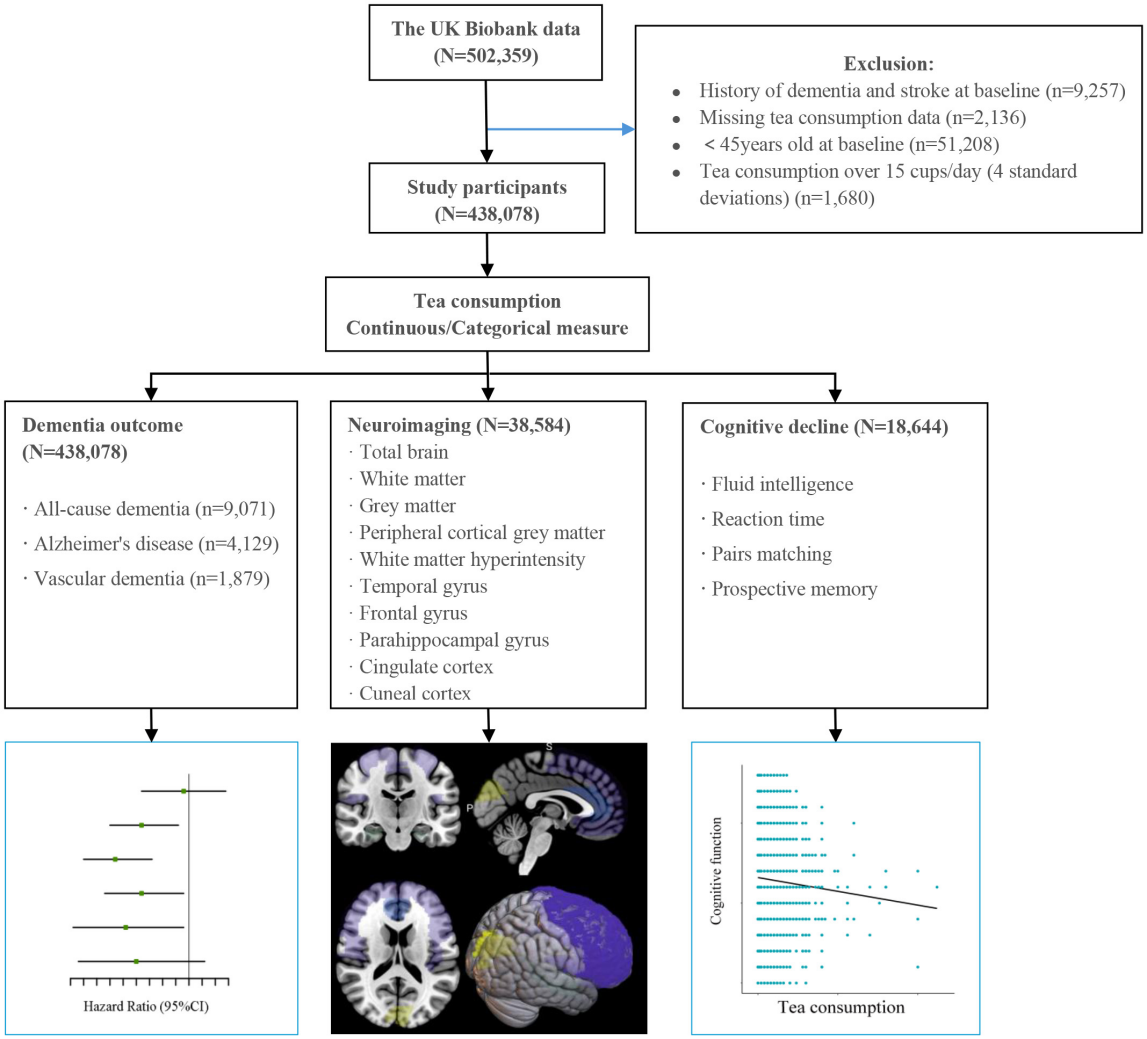
Flow diagram of the whole analysis process.

We excluded participants with prevalent dementia and stroke at baseline (*n* = 9,257), those with missing tea consumption data (*n* = 2,136), and exceptionally high values (>15 cups/day) for daily tea consumption (*n* = 1,680). Since dementia rarely occurs in young adults, we focused on individuals who were at least 45 years old at baseline. The final analysis dataset included 438,078 participants (Figure 1), of whom 38,584 had available brain imaging data (assessed from 2014 onward).

### Assessment of tea consumption

Tea consumption data were collected at baseline through the UK Biobank touchscreen questionnaire using a self-reported item (UKB Data-Field ID: 1488). Participants responded to the open-ended question: “How many cups of tea do you drink each day? (including black and green tea)open-ended que one of the following options: “Less than one”, “Do not know”, “Prefer not to answer”, or a specific number of cups of tea per day. If participants reported drinking more than 10 cups per day, they were asked to confirm their response. For analysis, participants were stratified into seven categories: non-drinkers, 0.5–1 1cups/day, 2–3 cups/day, 4–5 cups/day, 6–7 cups/day, 8–9 cups/day, and ≥ 10 cups/day.

### Neuroimaging outcomes

Brain imaging was performed using a Siemens Skyra 3 Tesla scanner (running VD13A SP4 software) with a standard 32-channel head coil. Previous literature (16, 17) has described brain MRI image acquisition and processing protocols in detail for the UK Biobank. In this study, we analyzed total brain volume, white matter, gray matter volume, white matter hyperintensities (WMH) volume, and regional gray matter volume (such as hippocampus, temporal pole, middle temporal gyrus, parahippocampal gyrus, superior frontal gyrus, frontal orbital cortex, frontal medial cortex, cuneal cortex, and cingulate cortex). The mean fractional anisotropy (FA) and mean diffusivity (MD) values of the weighted tracts in the cingulate and parahippocampal regions were also obtained. Mean FA and MD are two parameters in diffusion tensor imaging that quantitatively describe the integrity of white matter fibers, which are based on the diffusion of water molecules in brain tissue and characterize the geometric properties of the diffusion tensor (18).

The UK Biobank team used the Functional Magnetic Resonance Imaging of the Brain (FMRIB) software library (FSL; Version 5.0) to automate the pipeline preprocessing of the collected images (19). Total hippocampal volume was calculated as the sum of left and right hippocampal volumes. Due to right-skewed distributions, WMH volumes were log-transformed. All neuroimaging outcomes were standardized (mean = 0, standard deviation = 1) to facilitate comparison of effect sizes across outcomes. For multiple comparisons, the Benjamin-Hochberg procedure (the p.adjust function in R) was used to control the false discovery rate (FDR) at a set level of 5%.

### Dementia and its subtypes

Dementia was diagnosed according to inpatient records and death registries using the International Classification of Diseases, 10th revision (ICD-10) coding system. We chose the earliest recorded date of dementia code diagnosis regardless of the source. The primary outcomes in this study were incident all-cause dementia (ACD) and its 2 major component end points—Alzheimer's disease (AD) and vascular dementia (VaD), which were additionally analyzed separately. We defined outcomes according to the ICD-10 and self-reported (shown in Supplementary Table 1). The follow-up began on the date of attendance at the assessment center and continued until the earliest date of dementia diagnosis, loss to follow-up, death, or censoring (September 30, 2023), whichever occurred first.

### Assessment of cognitive function

UK Biobank participants completed four touchscreen cognitive tests at baseline and the first imaging visit (2014+), including (1) fluid intelligence (verbal-numerical reasoning), (2) pairs matching, (3) reaction time, and (4) prospective memory.

Fluid intelligence was assessed using a 13-item logic/reasoning questionnaire, with scores reflecting the maximum number of correct answers within 2 min. Pairs matching required participants to memorize the locations of six-card pairs within 3-s exposures, with scoring based on total false matches. Reaction time was measured through a symbolic matching task (similar to the “Snap” card game), calculated as mean response latency (milliseconds) across matching-pair trials. For prospective memory, participants were required to correctly recall the color/shape previously shown to them in the touch screen section, scoring 1 if remembering the first attempt and 0 if not. It has been reported that these cognitive tests have good concurrent validity and short-term stability (20, 21). Consistent with prior research (22), cognitive decline as a dichotomous variable was defined if fluid intelligence decreased by at least 1 point or reaction time increased by at least 100 ms. Based on the definition of fluid intelligence, cognitive decline as a dichotomous variables were defined if the total number of incorrect pairs matching increased by 1 point or if prospective memory accuracy decreased by at least 1 point.

### Assessment of covariates

In this study, we collected multiple potentially confounding variables, including demographic, lifestyle, and medical history factors, with a full description provided in Supplementary Table 1. Demographic variables comprised sex, age, ethnicity (White, mixed race, Asian, Black and other ethnic groups), qualification (college or university degree, Advanced [A] level/Advanced Subspecialty [AS] level or equivalent, Ordinary [O] level/General Certificate of Secondary Education [GCSE] or equivalent, Certificate of Secondary Education [CSE] or equivalent, National Vocational Qualification [NVQ] or Higher National Diploma [HND] or Higher National Certificate [HNC] or equivalent, other professional qualification, or none of the above), and income (less than £18,000, £18,000–£30,999, £31,000–£51,999, £52,000–£100,000, greater than £100,000). The Townsend deprivation index (TDI) was derived from the zip code of the area of residence, reflecting area-level socioeconomic deprivation based on car ownership, household overcrowding, home ownership, and unemployment (23), with higher scores indicating greater deprivation. Lifestyle factors included smoking and alcohol intake status (never, previous, and current), body mass index (BMI; calculated as weight in kilograms divided by height in meters squared), physical activity (light, moderate, and high), sleep duration; consumption of coffee, fruit, vegetable, and fish (yes and no). Medical history variables encompassed hypertension, cardiovascular disease (CVD), diabetes mellitus, cancer, depression status, and low-density lipoprotein cholesterol (LDL-C). LDL-C was measured using direct enzymatic methods (Kone lab, Thermo Fisher Scientific, Waltham, MA).

### Genetic risk factors

We evaluated the association between genetic risk factors for dementia, focusing on both apolipoprotein E (APOE) genotype and the non-APOE polygenic risk score (non-APOE PRS) for Alzheimer's disease (AD). Detailed information on these genetic risk factors can be found on the UK Biobank website. We stratified data according to APOE genotype (ε4 allele carriers vs. non-carriers) and non-APOE PRS (low, moderate, and high genetic risk groups by quintile). Only individuals with a self-reported white ethnic background were included in the polygenic risk score stratification analysis, and missing data were excluded.

### Statistical analysis

Baseline characteristics in the study were presented as numbers (percentages) for categorical variables and mean (standard deviation [SD]) for continuous variables, and were compared between groups using the Kruskal–Wallis rank-sum test and chi-square test. To account for the missing data of covariates, we used the random forest multiple imputation chain equation (24, 25) (method = ‘rf') from the ‘mice' package in R, and generated 5 imputed data sets to reduce sampling variability. Detailed information on missing covariates is shown in Supplementary Table 2. Each variable was missing in < 2% of participants, except for physical activity (23.46%), income (15.57%), and LDL-C (6.62%).

We investigated the association between tea consumption and neuroimaging outcomes using linear regression. Additionally, we assessed the relationship between tea consumption and dementia risk, and cognitive decline was analyzed using Cox regression and logistic regression, respectively. To explore potential non-linear relationships, we employed restricted cubic splines with tea consumption as a continuous variable. Non-tea drinkers served as the reference group. Two adjustment models were used: model 1 was partially adjusted for age, sex, and ethnicity; model 2 was fully adjusted for age, sex, ethnicity, education, Townsend deprivation index (TDI), body mass index (BMI), income, physical activity, sleep duration, smoking status, alcohol intake status, fish consumption, vegetables consumption, fruit consumption, coffee consumption, low-density lipoprotein cholesterol (LDL-C), depression status, hypertension, cardiovascular disease (CVD), diabetes, and cancer. Model 3 additionally included ICV as a covariate to control for its potential confounding influence on brain volume.

To examine the associations between tea consumption and neuroimaging outcomes as well as incident dementia, we performed seven sensitivity analyses. Firstly, we excluded participants with a history of chronic conditions (e.g., cardiovascular disease or cancer). Secondly, we excluded participants with a follow-up of less than 5 years. Thirdly, we repeated the analyses using a mixed-effects Cox regression model with assessment center as a random effect. Fourth, to investigate the potential modifying effect of the APOE genotype on the risk of dementia, we included APOE genotype (APOE ε4 carriers vs. non-carriers) as a covariate in the analysis. Fifth, given the impact of a healthy lifestyle on dementia, we included the healthy lifestyle score as a covariate in the analysis, as defined in previous literature (26). Sixth, we employed Schoenfeld's residual test for proportional hazards and identified significant violations for age and depression symptoms. To address this, we conducted sensitivity analysis by constructing time-dependent Cox models incorporating interaction terms between variables and follow-up time. Seventh, we repeated the analyses using the Fine-Gray competing risk model, with death considered as a competing event.

We also performed subgroup analyses stratified by age (midlife < 65 years, late-life ≥65 years) and sex. Moreover, Subgroup analyses were performed according to APOE genotype and non-APOE genetic risk score. We included two-way interaction terms between tea consumption and stratification factors and tested for significant interaction effects using likelihood ratio tests. FDR correction was applied only to the neuroimaging outcomes. All other analyses are exploratory, and their *p*-values are reported without adjustment for multiple comparisons. All statistical analyses and graphics were generated using R statistical software (version 4.2.3). A two-sided *p* < 0.05 was considered statistically significant.

## Results

### Baseline characteristics

Table 1 shows the baseline characteristics of the 438,078 participants (mean age of 58.11 [SD = 6.84] years, 55% female). The majority (95%) were White. Compared with non-tea drinkers, tea drinkers were more likely to be male, elderly, never smokers, and current alcohol drinkers, with no history of chronic diseases (e.g., cardiovascular disease, cancer). They also had higher education, income, and physical activities. As shown in Supplementary Table 3, the mean volumes of total brain, white matter, gray matter, and peripheral cortex were 1,151,676.34 (SD = 110,543.93) mm3, 542,951.95 (SD = 61,682.29) mm3, 608,724.41 (SD = 55,066.42) mm3, and 474,060.14 (SD = 45,461.07) mm3, respectively. During a median follow-up of 14.53 ([IQR] 13.69–15.25) years, there were 9,071 individuals diagnosed with dementia, which consisted of 4,129 AD and 1,879 VaD cases. Compared with participants without dementia, those with dementia were older, more economically deprived, less educated, more likely to smoke, less likely to drink alcohol, had higher BMI, and had smaller brain volumes. No significant differences were observed in coffee consumption, vegetable or fish intake, physical activity, or depressive status.

**Table 1:** Baseline characteristics by tea intake in the UK Biobank cohort.

**Variables**	**Overall**	**Tea intake, cups/day**							***p-*value**
		**0**	**0.5 to 1**	**2 to 3**	**4 to 5**	**6 to 7**	**8 to 9**	≥**10**	
Number of participants, *N* (%)	438,078	62,463 (14)	49,022 (11)	129,371 (30)	113,974 (26)	53,267 (12)	16,731 (4)	13,250 (3)	
Sex, male, *N* (%)	198,051 (45.2)	26,777 (42.9)	23,163 (47.3)	58,880 (45.5)	50,896 (44.7)	23,515 (44.1)	7,779 (46.5)	7,041 (53.1)	< 0.001
Age, Mean (SD), years	58.11 (6.84)	57.35 (6.91)	57.52 (6.96)	58.27 (6.86)	58.51 (6.76)	58.44 (6.70)	57.99 (6.77)	57.66 (6.79)	< 0.001
Ethnicity, *N* (%)									< 0.001
White	415,672 (95.2)	59,846 (96.2)	45,348 (92.9)	119,998 (93.1)	109,385 (96.3)	51,936 (97.8)	16,323 (97.9)	12,836 (97.3)	
Mixed	2,218 (0.5)	362 (0.6)	342 (0.7)	674 (0.5)	496 (0.4)	202 (0.4)	76 (0.5)	66 (0.5)	
Asian	9,006 (2.1)	661 (1.1)	1,266 (2.6)	4,422 (3.4)	1,946 (1.7)	463 (0.9)	118 (0.7)	130 (1.0)	
Black	6,131 (1.4)	953 (1.5)	1,217 (2.5)	2,435 (1.9)	1,093 (1.0)	267 (0.5)	79 (0.5)	87 (0.7)	
Others	3,525 (0.8)	409 (0.7)	616 (1.3)	1,399 (1.1)	713 (0.6)	241 (0.5)	71 (0.4)	76 (0.6)	
Education, *N* (%)									< 0.001
None of the above	79,221 (18.4)	11,310 (18.4)	6,184 (12.8)	21,200 (16.7)	22,063 (19.7)	11,300 (21.6)	3,822 (23.3)	3,342 (25.7)	
NVQ or HND or HNC	29,383 (6.8)	4,274 (7.0)	2,737 (5.7)	8,051 (6.3)	7,957 (7.1)	4,007 (7.7)	1,287 (7.8)	1,070 (8.2)	
CSEs or equivalent	21,335 (5.0)	3,529 (5.7)	1,962 (4.1)	5,894 (4.6)	5,567 (5.0)	2,801 (5.4)	831 (5.1)	751 (5.8)	
O levels/GCSEs	91,351 (21.2)	13,719 (22.3)	9,866 (20.5)	27,009 (21.3)	23,878 (21.3)	11,112 (21.3)	3,324 (20.3)	2,443 (18.8)	
A levels/AS levels	71,275 (16.6)	10,389 (16.9)	8,383 (17.4)	21,200 (16.7)	18,181 (16.3)	8,490 (16.2)	2,688 (16.4)	1,944 (15.0)	
College or University	137,663 (32.0)	18,208 (29.6)	19,093 (39.6)	43,677 (34.4)	34,208 (30.6)	14,577 (27.9)	4,450 (27.1)	3,450 (26.5)	
Income, *N* (%)									< 0.001
< 18k	86,962 (23.5)	12,845 (24.3)	8,225 (19.6)	23,511 (21.5)	23,041 (24.0)	11,768 (26.3)	4,031 (28.8)	3,541 (31.8)	
18k−31k	97,325 (26.3)	13,840 (26.2)	10,250 (24.4)	28,618 (26.2)	25,995 (27.1)	12,091 (27.0)	3,673 (26.2)	2,858 (25.6)	
31k−52k	94,885 (25.7)	13,517 (25.6)	11,199 (26.6)	28,525 (26.1)	24,535 (25.6)	11,145 (24.9)	3,378 (24.1)	2,586 (23.2)	
52k−100k	71,836 (19.4)	10,063 (19.1)	9,360 (22.3)	22,334 (20.4)	17,826 (18.6)	8,046 (18.0)	2,445 (17.5)	1,762 (15.8)	
>100k	18,876 (5.1)	2,513 (4.8)	2,998 (7.1)	6,286 (5.8)	4,458 (4.7)	1,740 (3.9)	484 (3.5)	397 (3.6)	
TDI, Mean (SD)	−1.40 (3.04)	−1.18 (3.13)	−1.27 (3.12)	−1.46 (3.03)	−1.55 (2.95)	−1.48 (2.97)	−1.19 (3.14)	−0.83 (3.27)	< 0.001
Physical activity, *N* (%)									< 0.001
Low	61,714 (18.4)	9,813 (20.7)	7,616 (19.8)	17,630 (17.6)	15,307 (17.6)	7,142 (17.8)	2,345 (18.8)	1,861 (18.8)	
Moderate	137,189 (40.9)	18,729 (39.5)	16,047 (41.7)	42,275 (42.2)	35,437 (40.8)	16,240 (40.5)	4,842 (38.8)	3,619 (36.6)	
High	136,418 (40.7)	18,924 (39.9)	14,856 (38.6)	40,182 (40.1)	36,052 (41.5)	16,711 (41.7)	5,282 (42.4)	4,411 (44.6)	
Smoking status, *N* (%)									< 0.001
Never	237,206 (54.4)	32,031 (51.5)	26,364 (54.0)	72,278 (56.1)	63,449 (55.9)	28,910 (54.5)	8,362 (50.2)	5,812 (44.1)	
Previous	155,868 (35.7)	22,102 (35.5)	17,439 (35.7)	46,447 (36.0)	40,767 (35.9)	18,717 (35.3)	5,837 (35.0)	4,559 (34.6)	
Current	43,323 (9.9)	8,117 (13.0)	5,062 (10.4)	10,118 (7.9)	9,328 (8.2)	5,419 (10.2)	2,463 (14.8)	2,816 (21.4)	
Alcohol intake status, *N* (%)									< 0.001
Never	18,949 (4.3)	3,355 (5.4)	1,940 (4.0)	5,640 (4.4)	4,366 (3.8)	2,212 (4.2)	747 (4.5)	689 (5.2)	
Previous	15,511 (3.5)	3,175 (5.1)	1,447 (3.0)	3,452 (2.7)	3,630 (3.2)	2,067 (3.9)	858 (5.1)	882 (6.7)	
Current	403,139 (92.1)	55,861 (89.5)	45,578 (93.1)	120,122 (93.0)	105,877 (93.0)	48,936 (92.0)	15,107 (90.4)	11,658 (88.1)	
Coffee consumption, *N* (%)	343,714 (78.6)	53,226 (85.3)	44,467 (90.8)	108,768 (84.2)	84,918 (74.6)	34,803 (65.4)	9,938 (59.5)	7,594 (57.5)	< 0.001
Fish consumption, *N* (%)	421,806 (96.3)	59,027 (94.5)	47,223 (96.3)	125,014 (96.6)	110,432 (96.9)	51,449 (96.6)	16,083 (96.1)	12,578 (94.9)	< 0.001
Vegetable consumption, *N* (%)	429,685 (98.1)	60,529 (96.9)	48,177 (98.3)	127,398 (98.5)	112,189 (98.4)	52,302 (98.2)	16,333 (97.6)	12,757 (96.3)	< 0.001
Fruit consumption, *N* (%)	414,946 (94.7)	57,367 (91.8)	46,696 (95.3)	124,113 (95.9)	108,925 (95.6)	50,348 (94.5)	15,564 (93.0)	11,933 (90.1)	< 0.001
Sleep duration, Mean (SD), hours	7.15 (1.11)	7.11 (1.17)	7.14 (1.09)	7.16 (1.08)	7.18 (1.08)	7.16 (1.11)	7.13 (1.17)	7.11 (1.28)	< 0.001
BMI, Mean (SD), kg/m^2^	27.47 (4.76)	28.21 (5.28)	27.48 (4.85)	27.23 (4.64)	27.29 (4.58)	27.42 (4.63)	27.64 (4.79)	27.69 (4.79)	< 0.001
LDL-C, Mean (SD), mmol/L	3.59 (0.87)	3.62 (0.89)	3.60 (0.87)	3.59 (0.87)	3.59 (0.87)	3.57 (0.86)	3.56 (0.86)	3.55 (0.88)	< 0.001
Depression status, *N* (%)	50,668 (11.6)	7,458 (11.9)	5,265 (10.7)	13,908 (10.8)	12,945 (11.4)	6,729 (12.6)	2,387 (14.3)	1,976 (14.9)	< 0.001
Cardiovascular diseases, *N* (%)	25,339 (5.8)	3,681 (5.9)	2,535 (5.2)	7,133 (5.5)	6,720 (5.9)	3,195 (6.0)	1,116 (6.7)	959 (7.2)	< 0.001
Hypertension, *N* (%)	132,772 (30.3)	18,945 (30.3)	14,467 (29.5)	39,247 (30.3)	34,781 (30.5)	16,086 (30.2)	5,132 (30.7)	4,114 (31.0)	0.001
Diabetes, *N* (%)	27,971 (6.4)	4,800 (7.7)	3,279 (6.7)	8,181 (6.3)	6,697 (5.9)	3,035 (5.7)	1,048 (6.3)	931 (7.0)	< 0.001
Cancer, *N* (%)	52,828 (12.1)	7,503 (12.0)	5,547 (11.3)	15,404 (11.9)	14,055 (12.3)	6,622 (12.4)	2,106 (12.6)	1,591 (12.0)	< 0.001

These variables have missing data and are not reported here.

P-values are derived using either Kruskal–Wallis's rank sum test and Pearson's Chi-squared test.

SD, standard deviation; NVQ, National Vocational Qualification; HND, Higher National Diploma; HNC, Higher National Certificate; CSE, Certificate of Secondary Education; O, Ordinary; GCSE, General Certificate of Secondary Education; A, Advanced; AS, Advanced Subsidiary; TDI, Townsend deprivation index; BMI, body mass index (calculated as weight in kilograms divided by height in meters squared); LDL-C, low-density lipoprotein cholesterol; UK Biobank, United Kingdom Biobank.

### Association between tea consumption and neuroimaging outcomes

**Figure 2:**
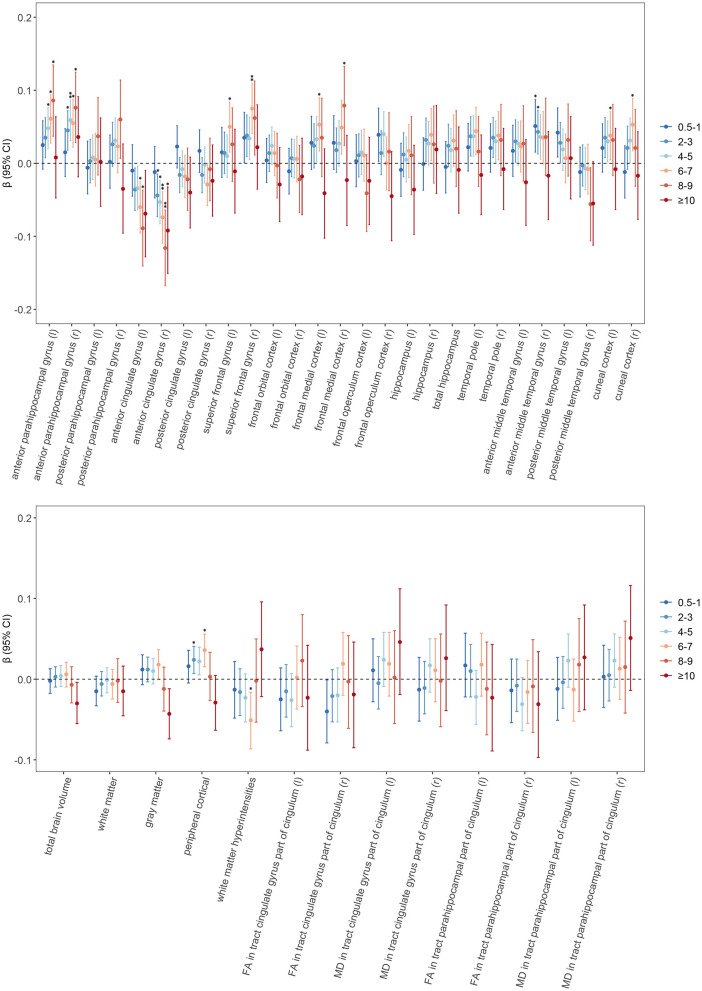
Associations of tea consumption with neuroimaging. General linear regression models were used to test for differences between different amounts of tea consumption and brain structure and white matter integrity. The circular points are the β values, and the error bars are their 95% confidence intervals. Asterisks denote statistically significant after FDR correction (*p < 0.05, **p < 0.01). Results were adjusted according to Model 3. The horizontal dashed line represents β of 0. Abbreviations FA, fractional anisotropy; MD, mean diffusivity; r, right; l, left; FDR, false discovery rate.

As shown in Figure 2 and Supplementary Table 4, Moderate tea consumption is significantly associated with the volume of specific brain regions (Model 1). After adjustment for multiple covariates (Model 2), the significance of some brain regions disappeared. Further adjustment by adding ICV (Model 3) showed that moderate tea consumption was significantly positively associated with the bilateral anterior parahippocampal gyrus. Drinking 6 to 7 cups of tea per day was positively associated with larger peripheral cortical volume (β = 0.036, *p*_FDR_ = 0.013), as well as with the bilateral superior frontal gyrus (left: β = 0.005, *p*_FDR_ = 0.039; right: β = 0.075, *p*_FDR_ = 0.002), left frontal medial cortex (β = 0.053, *p*_FDR_ = 0.039), and bilateral cuneal cortex volume (left: β = 0.038, *p*_FDR_ = 0.039; right: β = 0.053, *p*_FDR_ = 0.013). It was negatively associated with WMH volume (β = −0.051, *p*_FDR_ = 0.039) and the bilateral anterior cingulate gyrus (left: β = −0.060, *p*_FDR_ = 0.013; right: β = −0.074, *p*_FDR_ = 0.002). To elucidate the clinical significance of this effect, we converted the standardized effect size to raw units. The standardized estimate β = 0.069 implies that daily consumption of 4–5 cups of tea is associated with an average increase in total brain volume of approximately 7,628 mm3. Given that the mean total brain volume is 1,151,676 mm3, this difference corresponds to an increase in brain volume of approximately 0.66%. No significant associations were found with the hippocampus, temporal pole, and FA and MD values.

In three sensitivity analyses (Supplementary Tables 5–7), we found that the significant association between moderate tea consumption and brain volume remained. Subgroup analysis (Supplementary Table 8) showed that moderate tea consumption was significantly associated with larger anterior parahippocampal gyrus volume in males. This association was not found among women (Supplementary Table 9).

### Tea consumption and dementia outcome and cognitive function

**Figure 3:**
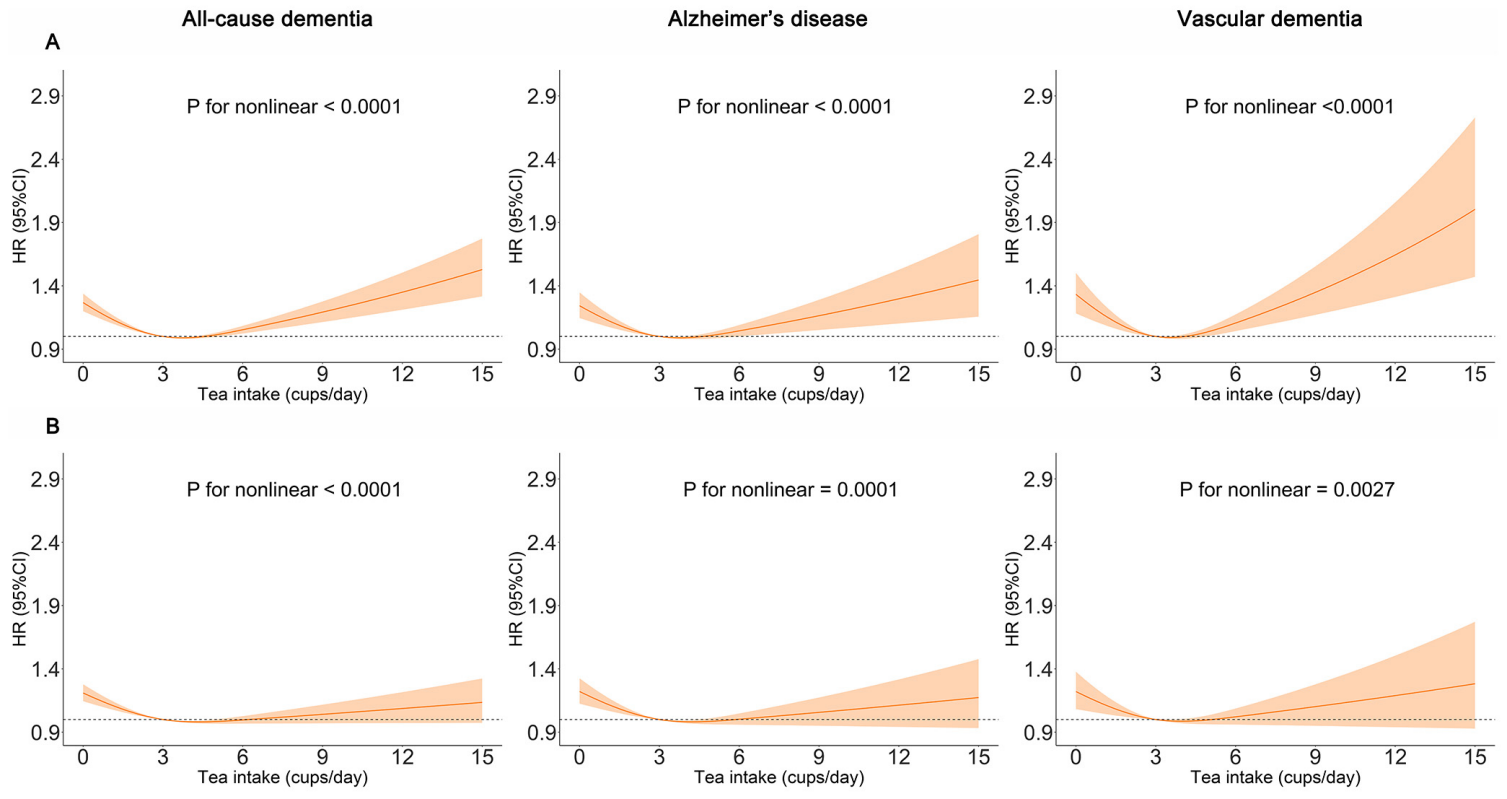
Restricted cubic spline models of the association between tea consumption and dementia. The 95% CIs of the adjusted HRs are represented by the shaded area. *P*-value was computed using restricted cubic spline functions in the Cox proportional hazard regression model. **(A)** Adjusted for age, sex, and ethnicity. **(B)** Adjusted for age, sex, ethnicity, Townsend deprivation index (TDI), education, income, body mass index (BMI), physical activity, sleep duration, smoking status, alcohol status, vegetables consumption, fruit consumption, fish consumption, coffee consumption, low-density lipoprotein cholesterol (LDL-C), Depression status, cardiovascular disease (CVD), hypertension, diabetes, cancer.

**Figure 4:**
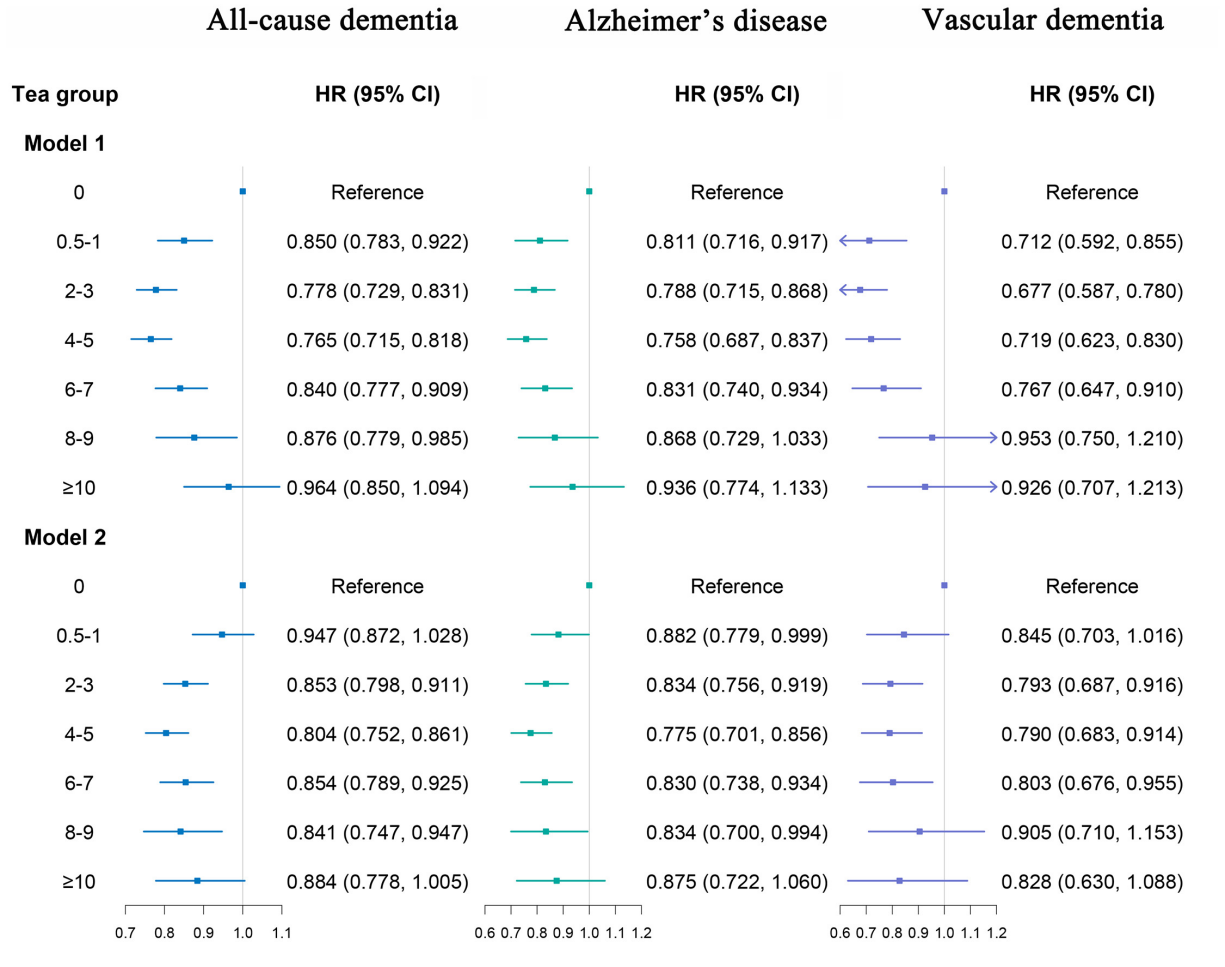
Association of tea consumption with dementia. Model 1: Adjusted for age, sex, and ethnicity. Model 2: Adjusted for age, sex, ethnicity, Townsend deprivation index (TDI), education, income, body mass index (BMI), physical activity, sleep duration, smoking status, alcohol status, vegetables consumption, fruit consumption, fish consumption, coffee consumption, low-density lipoprotein cholesterol (LDL-C), Depression status, cardiovascular disease (CVD), hypertension, diabetes, cancer. The *p*-values are unadjusted for multiple comparisons.

We found that moderate tea consumption was associated with a lower dementia risk. After adjusting for all covariates, the results remained unchanged. The association between tea consumption and dementia risk exhibited a non-linear pattern (*p* for non-linear < 0.0001) (Figure 3), with the consumption of 4 cups per day significantly related to a reduced risk of dementia. Individuals who consumed 4 to 5 cups of tea per day also had a lower risk of ACD (HR 0.804, 95% CI 0.752, 0.861, *p* < 0.001), AD (HR 0.775, 95% CI 0.701, 0.856, *p* < 0.001) and VaD (HR 0.790, 95% CI 0.683, 0.914, *p* = 0.002), compared with non-consumers (Figure 4, model 2). In seven additional sensitivity analyses (Supplementary Tables 10–16), the main results remained consistent. In subgroup analyses by sex and age (Supplementary Tables 17, 18), a significant association was only found in males (*p* for interaction < 0.05). There was no statistical significance between tea consumption and APOE genotype or non-APOE PRS (*p* for interaction > 0.05) (Supplementary Tables 19, 20). Furthermore, no significant associations were observed between tea consumption and cognitive decline (Table 2). It is important to note that these results have not been corrected for multiple comparisons and should be interpreted with caution.

**Table 2:** Association of tea consumption with cognitive decline.

**Tea (cups/day)**	**Fluid intelligence**		**Reaction time**		**Pairs matching**		**Prospective memory**	
	**OR (95% CI)**	***p*** **-value**	**OR (95% CI)**	***p*** **-value**	**OR (95% CI)**	***p*** **-value**	**OR (95% CI)**	***p*** **-value**
0	Reference		Reference		Reference		Reference	
0.5–1	1.110 (0.985, 1.250)	0.087	1.032 (0.906, 1.175)	0.640	0.815 (0.646, 1.029)	0.086	0.958 (0.707, 1.298)	0.783
2–3	1.029 (0.933, 1.134)	0.567	1.098 (0.988, 1.221)	0.082	0.908 (0.756, 1.091)	0.304	0.965 (0.756, 1.233)	0.778
4–5	1.020 (0.923, 1.127)	0.698	1.014 (0.909, 1.131)	0.803	0.864 (0.715, 1.046)	0.134	0.910 (0.707, 1.172)	0.466
6–7	1.051 (0.936, 1.179)	0.403	1.059 (0.934, 1.201)	0.369	0.841 (0.671, 1.054)	0.134	0.907 (0.676, 1.217)	0.514
8–9	0.935 (0.786, 1.112)	0.450	1.149 (0.956, 1.382)	0.140	0.978 (0.704, 1.357)	0.892	1.643 (1.139, 2.370)	0.008
≥10	1.022 (0.845, 1.236)	0.823	0.978 (0.794, 1.205)	0.837	1.111 (0.789, 1.565)	0.546	1.053 (0.674, 1.647)	0.819

Logistic regression model was used to analyze the association between different tea consumption and cognitive decline. Results were fully adjusted for all covariates.

OR < 1 represents poor cognitive function performance.

OR, odds ratio; CI, Confidence interval.

The p-values are unadjusted for multiple comparisons.

## Discussion

In this large-scale population study, we found moderate tea consumption was associated with larger volumes of the peripheral cortical gray matter, the anterior parahippocampal gyrus, cuneal cortex, and frontal lobe, as well as smaller volumes of WMH and the anterior cingulate cortex. These associations were most pronounced in males. The findings indicate that tea consumption could potentially mitigate brain atrophy and impede the progression of dementia.

Our results are consistent with a previous systematic review of cohort studies (27), which found that tea consumption was associated with reduced risk of dementia. He-Ying Hu et al. followed up 5,122 cases of all-cause dementia for 9 years using data from UKB and also found that moderate tea consumption (1–6 cups per day) was significantly associated with a reduced risk of dementia (28). In our study, we extended the follow-up period to 14.5 years and added approximately 4,000 cases of all-cause dementia. As the largest study to date examining this association, we provide more robust evidence that tea consumption is associated with all-cause dementia and its subtypes, with the association remaining consistent over more than 10 years of follow-up. In addition, we found a non-linear relationship between tea consumption and dementia risk, with a threshold effect. Consistent with previous studies (29, 30), we found that tea consumption was consistently associated with dementia regardless of genetic predisposition of APOE ε4 carrier status or non-APOE polygenic risk. Furthermore, we conducted several sensitivity analyses to test the stability of the results.

Our study found that moderate tea consumption was associated with larger volumes of the parahippocampal gyrus, cuneal cortex, and frontal lobe, which are linked to attenuation of neurodegeneration-prone regional atrophy. And these brain regions coincide with the core hubs of the default mode network (DMN), which point to cognitive function preservation (31). It's reported that potential tea supplementation was associated with the DMN in individuals with subjective memory impairment (32). Gray matter volume reduction, particularly in the parahippocampal gyrus, is a prominent feature during the transition from mild cognitive impairment to AD (33). A vast number of studies demonstrated links between established AD biomarkers and WMH, and the extent of WMH is correlated with cognitive impairment and an increased risk of dementia (34). Higher tea consumption was associated with fewer cerebral white matter lesions in older adults without dementia, suggesting that it may be useful in preventing dementia (35), which also supports our findings. Furthermore, we also observed a negative correlation between moderate tea consumption and the volume of the ACC. We hypothesize that this morphometric change may reflect a process of neural optimization (36) or habitual preference in emotional regulation within the ACC (37).

Similar to a previous study (38), we did not observe a significant association between tea consumption and cognitive decline. This discrepancy may be related to the relatively healthy status of the UK Biobank participants, the smaller sample size, and the short follow-up period. Further longitudinal studies in diverse populations are needed to assess whether the relationship between tea consumption, dementia-related biomarkers, and cognitive performance changes with age and disease progression.

Numerous studies (39–41) have suggested that diet or dietary patterns may affect brain structure, thereby playing a role in dementia intervention. We speculate that the neuroprotective associations observed in our cohort, where black tea consumption was predominant, may be attributable to the maintenance of brain volume. However, due to the paucity of data regarding tea types, the mechanistic attribution to specific tea compounds remains conjectural. Research on black tea consumption suggests that specific compounds, notably theaflavins, may have neuroprotective effects (42), particularly on cognitive function (42). To our knowledge, no previous study has investigated the association of tea consumption with dementia-related brain biomarkers. Larger longitudinal studies are needed to validate these findings and examine the effects of individual tea components.

Our study observed a sex-specific association between tea consumption and brain health, with the association being more pronounced in men than in women. This observed difference aligns with established evidence of sexual dimorphism in brain structure and function during aging (43). The underlying reasons for this disparity remain unclear from our data, as we did not measure specific tea types or constituents. However, previous studies have indicated that estrogen influences multiple neural pathways (44), and its decline in postmenopausal women is linked to changes in brain metabolism and the integrity of neural systems such as the cholinergic system (45). The observed sex-specific associations are consistent with the known biological differences between sexes, which may provide a plausible context for interpreting these findings. The presence of such sex-specific associations underscores the importance of considering sex as a biological variable in future studies on tea consumption and brain health.

Strengths of this study include the large sample size cohort from UKB, long-term follow-up, adjustment for multiple confounding factors, and investigation of the association between tea consumption and neuroimaging markers related to dementia. However, there are still some limitations that need to be addressed. Firstly, the cross-sectional nature of our neuroimaging outcomes limits causal inference. We cannot exclude the possibility that subtle, unmeasured precursors of brain health may influence tea-drinking behavior. Therefore, the results should be interpreted with caution. Future studies with longitudinal designs are needed to confirm the direction of the observed associations. Secondly, over 80% of participants in this study maintained stable tea-drinking habits. However, the tea consumption data were based on self-reports without standardized cup sizes or differentiation between tea types, and lacked dynamic changes. These limitations may have affected the precise estimation of the dose–response relationship and weakened causal inference. Notably, black tea accounts for over 80% of total tea consumption in the UK (46), so the tea consumption data in the UK Biobank questionnaire mainly reflect black tea, which aligns with local cultural practices. Future research should use prospective designs and standardized measurement methods for further validation. Thirdly, despite incorporating a healthy lifestyle score to capture overall health behavior patterns, tea consumption may still be associated with other unmeasured factors. Moreover, the observational design cannot completely rule out the possibility of reverse causality. Additionally, the exploratory nature of our non-neuroimaging analyses and the lack of multiple testing correction mean that these findings should be interpreted as preliminary and require future validation. Fourth, since the study population was predominantly white British, the results may be influenced by race-specific metabolic differences. Although genetic stratification analysis did not reveal significant interactions, it still requires validation in ethnically diverse populations in the future. Finally, due to the selection bias of healthy volunteers in the UK Biobank (15), the association between tea drinking and dementia found in this study may be more applicable to populations with high health awareness and high socioeconomic status. Further validation is needed in more representative population cohorts.

## Conclusions

In conclusion, our study provides new evidence that moderate tea consumption is associated with neurodegeneration and dementia-related brain markers. These findings highlight the significance of moderate tea intake in maintaining brain health, offering valuable guidance for developing preventive measures and dietary intervention strategies against dementia.

## Data Availability

The original contributions presented in the study are included in the article/Supplementary material, further inquiries can be directed to the corresponding authors.
