# Supplementary material for: Moderate tea consumption and dementia-related neuroimaging markers
Source: Front Neurol. 2025 Nov 11;16:1634621. doi: 10.3389/fneur.2025.1634621 (PMC12644034; doi:10.3389/fneur.2025.1634621)
Supplement: Supplementary file 1 [file Table_1.docx]

| **Diseases** | **Self-reported** | **ICD-9** | **ICD-10** | **OPCS-4** | **Examination** |
| --- | --- | --- | --- | --- | --- |
| All-cause dementia | 42018, 20002 (1263) |  | 41270 (G30, G30.0, G30.1, G30.8, G30.9, G31.0, G31.1, G31.8, F00, F00.0, F00.1, F00.2, F00.9, F01, F01.0, F01.1, F01.2, F01.3, F01.8, F01.9, F02, F02.0, F02.1, F02.2, F02.3, F02.4, F02.8, F03, F05.1, F10.6, A81.0, I67.3) |  |  |
| Alzheimer's disease | 42020 |  | 41270 (G30.0, G30.1, G30.8, G30.9, F00, F00.0, F00.1, F00.2, F00.9) |  |  |
| Vascular dementia | 42022 |  | 41270 (F01.0, F01.2, F01.3, F01.8, F01.9, I67.3) |  |  |
| Hypertension | 6150 (4), 2966, 6153 (2), 6177 (2), 20002 (1065, 1072) | 41271 (401-405) | 41270 (I10, I11, I12, I13, I15, O10, O11) |  |  |
| Cardiovascular Disease | 6150 (1,2), 3894, 4056, 3627, 20002 (1074, 1075, 1081, 1583, 1086, 1491) | 41271 (410, 411, 412, 413, 414, 430, 431, 434, 436) | 41270 (I20, I21, I22, I23, I24, I25, I60, I61, I62, I63, I64, Z951, Z955) | 41272 (K40, K41, K42, K43, K45, K46, K49, K50, K75) |  |
| Diabetes | 2443 (1), 2976, 6153 (3), 6177 (3), 20002 (1220, 1222, 1223) | 41271 (250, 3572, 3620) | 41270 (E10, E11, E12, E13, E14, G590, G632, H280, H360, M142, N083) |  | 30740, 30750 |
| Cancer | 20001, 40005, 2453 | 41271(140-208) | 41270 (C00-C97) |  |  |
| Depression status | 20123, 20124, 20125,  20002 (1286, 1531) |  | 41270 (F32-34, F38-39) |  |  |
| Stroke | 6150 (3), 4056, 42006, 42008, 42010, 42012,20002 (1081, 1491, 1583, 1086) | 41271 (430, 431, 434, 436) | 41270 (I60, I61, I62.9, I63, I64, I67.8, I69.0, I69.3, G951, H341, H342, S066) | 41272 (A052, A053, A054, L351, L353, L343) |  |

**Supplementary Table 1.** Codes for international classification disease and self-reported fields for different diseases

Abbreviations: ICD, International Classification of Diseases; OPCS-4, Office of Population Censuses and Surveys Classification of Interventions and Procedures, version 4.

**Supplementary Table 2.** Detailed information on missing covariates.

| **Covariates** | **Number** | **Missing rate (%)** |
| --- | --- | --- |
| Education | 7,850 | 1.79 |
| Ethnicity | 1,526 | 0.35 |
| Income | 68,194 | 15.57 |
| Townsend deprivation index | 490 | 0.11 |
| Physical activity | 102,757 | 23.46 |
| Smoking status | 1,681 | 0.38 |
| Alcohol intake status | 479 | 0.11 |
| Coffee consumption | 696 | 0.16 |
| Sleep duration | 2,577 | 0.59 |
| BMI | 2,236 | 0.51 |
| LDL-C | 28,996 | 6.62 |

Abbreviations: BMI, body mass index (calculated as weight in kilograms divided by height in meters squared); LDL-C, low-density lipoprotein cholesterol.

**Supplementary Table 3.** Baseline characteristics of study participants by dementia.

| **Variables** | **Overall** | **No incident dementia** | **Incident dementia** | ***p* value** |
| --- | --- | --- | --- | --- |
| Number of participants | 438,078 | 429,007 | 9,071 |  |
| Follow up time, Median (IQR) | 14.53 (13.69, 15.25) | 14.55 (13.79, 15.27) | 10.64 (8.20, 12.39) | < 0.001 |
| Age, years, Mean (SD) | 58.11 (6.84) | 57.98 (6.82) | 64.30 (4.50) | < 0.001 |
| Sex, male, N (%) | 198,051 (45.2) | 193,398 (45.1) | 4,653 (51.3) | < 0.001 |
| Tea consumption, N (%) |  |  |  | < 0.001 |
| Non-consumption | 62,463 (14) | 61,095 (14) | 1,368 (15) |  |
| Consumption | 375,615 (86) | 367,912 (86) | 7,703 (85) |  |
| Ethnicity, N (%) |  |  |  | < 0.001 |
| White | 415,672 (95.2) | 406,988 (95.2) | 8,684 (96.1) |  |
| Mixed | 2,218 (0.5) | 2,187 (0.5) | 31 (0.3) |  |
| Asian | 9,006 (2.1) | 8,868 (2.1) | 138 (1.5) |  |
| Black | 6,131 (1.4) | 6,002 (1.4) | 129 (1.4) |  |
| Other | 3,525 (0.8) | 3,469 (0.8) | 56 (0.6) |  |
| Education, N (%) |  |  |  | < 0.001 |
| None of the above | 79,221 (18.4) | 76,165 (18.1) | 3,056 (35.0) |  |
| NVQ or HND or HNC | 29,383 (6.8) | 28,728 (6.8) | 655 (7.5) |  |
| CSEs or equivalent | 21,335 (5.0) | 21,125 (5.0) | 210 (2.4) |  |
| O levels/GCSEs | 91,351 (21.2) | 89,684 (21.3) | 1,667 (19.1) |  |
| A levels/AS levels | 71,275 (16.6) | 70,000 (16.6) | 1,275 (14.6) |  |
| College or University | 137,663 (32.0) | 135,785 (32.2) | 1,878 (21.5) |  |
| Townsend deprivation index, Mean (SD) | -1.40 (3.04) | -1.40 (3.04) | -1.05 (3.25) | < 0.001 |
| Income, N (%) |  |  |  | < 0.001 |
| <18k | 86,962 (23.5) | 83,907 (23.1) | 3,055 (44.4) |  |
| 18k-31k | 97,325 (26.3) | 95,203 (26.2) | 2,122 (30.9) |  |
| 31k-52k | 94,885 (25.7) | 93,786 (25.8) | 1,099 (16.0) |  |
| 52k-100k | 71,836 (19.4) | 71,349 (19.7) | 487 (7.1) |  |
| >100k | 18,876 (5.1) | 18,764 (5.2) | 112 (1.6) |  |
| Physical activity, N (%) |  |  |  | 0.578 |
| Low | 61,714 (18.4) | 60,575 (18.4) | 1,139 (18.4) |  |
| Moderate | 137,189 (40.9) | 134,619 (40.9) | 2,570 (41.5) |  |
| High | 136,418 (40.7) | 133,936 (40.7) | 2,482 (40.1) |  |
| Smoking status, N (%) |  |  |  | < 0.001 |
| Never | 237,206 (54.4) | 232,952 (54.5) | 4,254 (47.2) |  |
| Previous | 155,868 (35.7) | 152,035 (35.6) | 3,833 (42.6) |  |
| Current | 43,323 (9.9) | 42,403 (9.9) | 920 (10.2) |  |
| Alcohol intake status, N (%) |  |  |  | < 0.001 |
| Never | 18,949 (4.3) | 18,336 (4.3) | 613 (6.8) |  |
| Previous | 15,511 (3.5) | 14,946 (3.5) | 565 (6.2) |  |
| Current | 403,139 (92.1) | 395,266 (92.2) | 7,873 (87.0) |  |
| Sleep duration, Mean (SD), hours | 7.15 (1.11) | 7.15 (1.10) | 7.24 (1.29) | < 0.001 |
| BMI, Mean (SD), kg/m^2^ | 27.47 (4.76) | 27.46 (4.76) | 27.72 (4.89) | < 0.001 |
| LDL_C, Mean (SD), mmol/L | 3.59 (0.87) | 3.59 (0.87) | 3.48 (0.96) | < 0.001 |
| Coffee consumption, N (%) | 343,714 (78.6) | 336,594 (78.6) | 7,120 (78.7) | 0.765 |
| Vegetable consumption, N (%) | 429,685 (98.1) | 420,893 (98.1) | 8,792 (96.9) | < 0.001 |
| Fruit consumption, N (%) | 414,946 (94.7) | 406,387 (94.7) | 8,559 (94.4) | 0.123 |
| Fish consumption, N (%) | 421,806 (96.3) | 413,062 (96.3) | 8,744 (96.4) | 0.597 |
| Depression status, N (%) | 50,668 (11.6) | 49,578 (11.6) | 1,090 (12.0) | 0.181 |
| Cardiovascular diseases, N (%) | 25,339 (5.8) | 24,017 (5.6) | 1,322 (14.6) | < 0.001 |
| Hypertension, N (%) | 132,772 (30.3) | 128,655 (30.0) | 4,117 (45.4) | < 0.001 |
| Diabetes, N (%) | 27,971 (6.4) | 26,677 (6.2) | 1,294 (14.3) | < 0.001 |
| Cancer, N (%) | 52,828 (12.1) | 51,471 (12.0) | 1,357 (15.0) | < 0.001 |
| APOE *ε*4 carriers, N (%) | 102,512 (24.1) | 98,543 (23.6) | 3,969 (45.5) | < 0.001 |
| Non-APOE PRS, N (%) |  |  |  | <0.001 |
| Low | 84817 (19.4) | 83899 (19.6) | 918 (10.1) |  |
| Intermediate | 254462 (58.1) | 250376 (58.4) | 4086 (45.0) |  |
| High | 98799 (22.6) | 94732 (22.1) | 4067 (44.8) |  |
| Total brain volume, mm^3^ Mean (SD) | 1,151,676.34 (110,543.93) | 1,151,808.27 (110,510.64) | 1,115,027.71 (114,042.50) | < 0.001 |
| Volume of white matter, mm^3^ Mean (SD) | 542,951.95 (61,682.29) | 542,959.31 (61,645.07) | 540,906.21 (71,488.56) | 0.688 |
| Volume of grey matter, mm^3^ Mean (SD) | 608,724.41 (55,066.42) | 608,848.98 (55,034.19) | 574,121.14 (53,249.95) | < 0.001 |
| Volume of peripheral cortical grey matter, mm^3^ Mean (SD) | 474,060.14 (45,461.07) | 474,168.89 (45,431.72) | 443,850.53 (43,647.11) | < 0.001 |

These variables have missing data and are not reported here.

*P*-values are derived using either Wilcoxon rank sum test or chi-square test.

Abbreviations: IQR Inter-quartile range. SD, standard deviation; NVQ, National Vocational Qualification; HND, Higher National Diploma; HNC, Higher National Certificate; CSE, Certificate of Secondary Education; O, Ordinary; GCSE, General Certificate of Secondary Education; A, Advanced; AS, Advanced Subsidiary; BMI, body mass index (calculated as weight in kilograms divided by height in meters squared); LDL-C, low-density lipoprotein Cholesterol; UK Biobank, United Kingdom Biobank; APOE, apolipoprotein E; Low (lowest quintile of PRS), Intermediate (PRS quintile 2 to 4), High ( highest quintile of PRS). PRS, polygenic risk score.

**Supplementary Table 10.** Association of tea consumption with dementia after exclusion of individuals with major prior diseases (e.g., CVD and cancer) (n = 363,296).

| **Tea (cups/day)** | **All-cause dementia** | | **Alzheimer’s disease** | | **Vascular dementia** | |
| --- | --- | --- | --- | --- | --- | --- |
|  | **HR (95% CI)** | ***p* value** | **HR (95% CI)** | ***p* value** | **HR (95% CI)** | ***p* value** |
| **Model 1** |  |  |  |  |  |  |
| 0 | Reference |  | Reference |  | Reference |  |
| 0.5-1 | 0.904 (0.821, 0.994) | **0.038** | 0.841 (0.729, 0.969) | **0.016** | 0.740 (0.592, 0.926) | **0.008** |
| 2-3 | 0.804 (0.744, 0.869) | **<0.001** | 0.817 (0.731, 0.914) | **<0.001** | 0.694 (0.582, 0.827) | **<0.001** |
| 4-5 | 0.786 (0.726, 0.851) | **<0.001** | 0.771 (0.687, 0.865) | **<0.001** | 0.736 (0.616, 0.878) | **0.001** |
| 6-7 | 0.859 (0.783, 0.943) | **0.001** | 0.809 (0.705, 0.927) | **0.002** | 0.777 (0.629, 0.959) | **0.019** |
| 8-9 | 0.881 (0.765, 1.013) | 0.076 | 0.868 (0.707, 1.065) | 0.176 | 0.944 (0.698, 1.270) | 0.708 |
| ≥10 | 1.007 (0.867, 1.169) | 0.929 | 1.034 (0.834, 1.283) | 0.759 | 1.008 (0.727, 1.399) | 0.960 |
| **Model 2** |  |  |  |  |  |  |
| 0 | Reference |  | Reference |  | Reference |  |
| 0.5-1 | 0.990 (0.899, 1.090) | 0.834 | 0.905 (0.784, 1.043) | 0.168 | 0.851 (0.680, 1.066) | 0.160 |
| 2-3 | 0.871 (0.806, 0.942) | **0.001** | 0.859 (0.767, 0.961) | **0.008** | 0.801 (0.671, 0.957) | **0.014** |
| 4-5 | 0.822 (0.758, 0.890) | **<0.001** | 0.783 (0.697, 0.880) | **<0.001** | 0.809 (0.676, 0.969) | **0.021** |
| 6-7 | 0.869 (0.791, 0.955) | **0.004** | 0.802 (0.698, 0.921) | **0.002** | 0.816 (0.659, 1.012) | 0.064 |
| 8-9 | 0.859 (0.745, 0.990) | **0.035** | 0.840 (0.683, 1.033) | 0.099 | 0.941 (0.693, 1.276) | 0.694 |
| ≥10 | 0.934 (0.804, 1.086) | 0.375 | 0.969 (0.779, 1.204) | 0.774 | 0.948 (0.681, 1.320) | 0.752 |

Model 1: Adjusted for age, sex, and ethnicity.

Model 2: Adjusted for age, sex, ethnicity, education, Townsend deprivation index, body mass index, income, physical activity, sleep duration, smoking status, alcohol intake status, fish consumption, vegetable consumption, fruit consumption, coffee consumption, low-density lipoprotein cholesterol, depression status, hypertension, cardiovascular diseases, diabetes, and cancer.

HR, hazard ratios; CI, confidence interval.

Bold indicates statistical significance (*p*-value < 0.05). The *p*-values are unadjusted for multiple comparisons.

**Supplementary Table 11.** Risk of dementia in participants consuming different cups of tea in sensitivity analysis restricting individuals to those with the follow-up time of ≥5 years (n = 428,960).

| **Tea (cups/day)** | **All-cause dementia** | | **Alzheimer’s disease** | | **Vascular dementia** | |
| --- | --- | --- | --- | --- | --- | --- |
|  | **HR (95% CI)** | ***p* value** | **HR (95% CI)** | ***p* value** | **HR (95% CI)** | ***p* value** |
| **Model 1** |  |  |  |  |  |  |
| 0 | Reference |  | Reference |  | Reference |  |
| 0.5-1 | 0.854 (0.784, 0.931) | **<0.001** | 0.825 (0.725, 0.937) | **0.003** | 0.734 (0.606, 0.888) | **0.001** |
| 2-3 | 0.778 (0.727, 0.833) | **<0.001** | 0.803 (0.726, 0.888) | **<0.001** | 0.670 (0.577, 0.778) | **<0.001** |
| 4-5 | 0.765 (0.713, 0.820) | **<0.001** | 0.766 (0.691, 0.850) | **<0.001** | 0.708 (0.609, 0.824) | **<0.001** |
| 6-7 | 0.844 (0.778, 0.916) | **<0.001** | 0.842 (0.746, 0.950) | **0.005** | 0.802 (0.672, 0.956) | **0.014** |
| 8-9 | 0.872 (0.772, 0.986) | **0.029** | 0.853 (0.710, 1.025) | 0.089 | 0.964 (0.751, 1.239) | 0.776 |
| ≥10 | 0.980 (0.860, 1.117) | 0.764 | 0.969 (0.797, 1.179) | 0.755 | 0.944 (0.713, 1.251) | 0.689 |
| **Model 2** |  |  |  |  |  |  |
| 0 | Reference |  | Reference |  | Reference |  |
| 0.5-1 | 0.951 (0.872, 1.036) | 0.248 | 0.898 (0.789, 1.021) | 0.101 | 0.869 (0.717, 1.052) | 0.151 |
| 2-3 | 0.851 (0.795, 0.912) | **<0.001** | 0.849 (0.767, 0.940) | **0.002** | 0.784 (0.674, 0.912) | **0.002** |
| 4-5 | 0.803 (0.748, 0.862) | **<0.001** | 0.782 (0.705, 0.869) | **<0.001** | 0.781 (0.670, 0.911) | **0.002** |
| 6-7 | 0.857 (0.789, 0.931) | **<0.001** | 0.839 (0.742, 0.949) | **0.005** | 0.844 (0.706, 1.011) | 0.065 |
| 8-9 | 0.838 (0.740, 0.948) | **0.005** | 0.819 (0.681, 0.985) | **0.034** | 0.928 (0.720, 1.196) | 0.565 |
| ≥10 | 0.901 (0.789, 1.028) | 0.122 | 0.904 (0.742, 1.102) | 0.320 | 0.856 (0.643, 1.138) | 0.283 |

Model 1: Adjusted for age, sex, and ethnicity.

Model 2: Adjusted for age, sex, ethnicity, education, Townsend deprivation index, body mass index, income, physical activity, sleep duration, smoking status, alcohol intake status, coffee consumption, fish consumption, vegetable consumption, fruit consumption, low-density lipoprotein cholesterol, depression status, hypertension, cardiovascular diseases, diabetes, and cancer.

Bold indicates statistical significance (*p*-value < 0.05). The *p*-values are unadjusted for multiple comparisons.

**Supplementary Table 12.** Association of tea consumption with dementia in sensitivity analysis using mixed effects Cox regression (assessment center as a random effect) (n = 438,078).

| **Tea (cups/day)** | **All-cause dementia** | | **Alzheimer’s disease** | | **Vascular dementia** | |
| --- | --- | --- | --- | --- | --- | --- |
|  | **HR (95% CI)** | ***p* value** | **HR (95% CI)** | ***p* value** | **HR (95% CI)** | ***p* value** |
| **Model 1** |  |  |  |  |  |  |
| 0 | Reference |  | Reference |  | Reference |  |
| 0.5-1 | 0.852 (0.785, 0.925) | **<0.001** | 0.812 (0.717, 0.918) | **0.001** | 0.723 (0.602, 0.868) | **0.001** |
| 2-3 | 0.779 (0.730, 0.832) | **<0.001** | 0.788 (0.716, 0.868) | **<0.001** | 0.684 (0.593, 0.788) | **<0.001** |
| 4-5 | 0.764 (0.714, 0.817) | **<0.001** | 0.758 (0.687, 0.837) | **<0.001** | 0.717 (0.622, 0.828) | **<0.001** |
| 6-7 | 0.838 (0.775, 0.907) | **<0.001** | 0.831 (0.740, 0.934) | **0.002** | 0.760 (0.641, 0.900) | **0.002** |
| 8-9 | 0.874 (0.777, 0.983) | **0.025** | 0.868 (0.729, 1.033) | 0.110 | 0.942 (0.741, 1.197) | 0.624 |
| ≥10 | 0.962 (0.848, 1.092) | 0.550 | 0.938 (0.776, 1.135) | 0.511 | 0.917 (0.700, 1.201) | 0.527 |
| **Model 2** |  |  |  |  |  |  |
| 0 | Reference |  | Reference |  | Reference |  |
| 0.5-1 | 0.949 (0.874, 1.031) | 0.214 | 0.882 (0.779, 0.999) | **0.047** | 0.854 (0.710, 1.026) | 0.092 |
| 2-3 | 0.852 (0.798, 0.911) | **<0.001** | 0.832 (0.755, 0.917) | **<0.001** | 0.796 (0.690, 0.919) | **0.002** |
| 4-5 | 0.805 (0.752, 0.862) | **<0.001** | 0.776 (0.702, 0.858) | **<0.001** | 0.790 (0.683, 0.914) | **0.002** |
| 6-7 | 0.855 (0.789, 0.926) | **<0.001** | 0.833 (0.740, 0.937) | **0.002** | 0.801 (0.673, 0.952) | **0.012** |
| 8-9 | 0.841 (0.747, 0.947) | **0.004** | 0.835 (0.701, 0.996) | **0.045** | 0.902 (0.708, 1.150) | 0.405 |
| ≥10 | 0.885 (0.779, 1.006) | 0.061 | 0.878 (0.724, 1.064) | 0.183 | 0.826 (0.628, 1.085) | 0.170 |

Model 1: Adjusted for age, sex, and ethnicity.

Model 2: Adjusted for age, sex, ethnicity, education, Townsend deprivation index, body mass index, income, physical activity, sleep duration, smoking status, alcohol intake status, coffee consumption, fish consumption, vegetable consumption, fruit consumption, low-density lipoprotein cholesterol, depression status, hypertension, cardiovascular diseases, diabetes, and cancer.

Bold indicates statistical significance (*p*-value < 0.05). The *p*-values are unadjusted for multiple comparisons.

**Supplementary Table 13.** Association of tea consumption with dementia in sensitivity analysis with adjusting APOE genotype (n = 438,078).

| **Tea (cups/day)** | **HR (95% CI)** | ***p* value** |
| --- | --- | --- |
| 0 | Reference |  |
| 0.5-1 | 0.938 (0.864, 1.019) | 0.132 |
| 2-3 | 0.850 (0.796, 0.908) | **<0.001** |
| 4-5 | 0.801 (0.748, 0.857) | **<0.001** |
| 6-7 | 0.849 (0.784, 0.919) | **<0.001** |
| 8-9 | 0.831 (0.738, 0.936) | **0.002** |
| ≥10 | 0.890 (0.784, 1.012) | 0.075 |

Model adjusted for age, sex, ethnicity, education, Townsend deprivation index, body mass index, income, physical activity, sleep duration, smoking status, alcohol intake status, coffee consumption, fish consumption, vegetable consumption, fruit consumption, low-density lipoprotein cholesterol, depression status, hypertension, cardiovascular diseases, diabetes, and cancer, APOE genotype.

Bold indicates statistical significance (*p*-value < 0.05). APOE, apolipoprotein E. The *p*-values are unadjusted for multiple comparisons.

**Supplementary Table 14.** Association of tea consumption with dementia in sensitivity analysis with adjusting healthy lifestyle score. (n=438,078)

| **Tea (cups/day)** | **HR (95% CI)** | ***p* value** |
| --- | --- | --- |
| 0 | Reference |  |
| 0.5-1 | 0.947 (0.872, 1.029) | 0.199 |
| 2-3 | 0.853 (0.799, 0.911) | **<0.001** |
| 4-5 | 0.805 (0.752, 0.861) | **<0.001** |
| 6-7 | 0.854 (0.789, 0.925) | **<0.001** |
| 8-9 | 0.841 (0.747, 0.947) | **0.004** |
| ≥10 | 0.884 (0.778, 1.004) | 0.058 |

Model adjusted for age, sex, ethnicity, education, Townsend deprivation index, body mass index, income, physical activity, sleep duration, smoking status, alcohol intake status, coffee consumption, fish consumption, vegetable consumption, fruit consumption, low-density lipoprotein cholesterol, depression status, hypertension, cardiovascular diseases, diabetes, cancer, and healthy lifestyle score

Bold indicates statistical significance (*p*-value < 0.05). The *p*-values are unadjusted for multiple comparisons.

**Supplementary Table 15.** Association of tea consumption with dementia in sensitivity analysis accounting for time-varying effects of age and depression. (n=438,078)

| **Tea (cups/day)** | **HR (95% CI)** | ***p* value** |
| --- | --- | --- |
| 0 | Reference |  |
| 0.5-1 | 0.980 (0.902, 1.064) | 0.628 |
| 2-3 | 0.872 (0.816, 0.932) | **<0.001** |
| 4-5 | 0.809 (0.756, 0.866) | **<0.001** |
| 6-7 | 0.851 (0.786, 0.922) | **<0.001** |
| 8-9 | 0.828 (0.735, 0.932) | **0.002** |
| ≥10 | 0.860 (0.757, 0.977) | 0.021 |

Model adjusted for age, sex, ethnicity, education, Townsend deprivation index, body mass index, income, physical activity, sleep duration, smoking status, alcohol intake status, coffee consumption, fish consumption, vegetable consumption, fruit consumption, low-density lipoprotein cholesterol, depression status, hypertension, cardiovascular diseases, diabetes, cancer.

Bold indicates statistical significance (*p*-value < 0.05). The *p*-values are unadjusted for multiple comparisons.

**Supplementary Table 16.** Association between tea consumption and dementia using a competing risk model. (n=438,078)

| **Tea (cups/day)** | **Model 1** | | **Model 2** | |
| --- | --- | --- | --- | --- |
|  | **HR (95% CI)** | ***p* value** | **HR (95% CI)** | ***p* value** |
| 0 | Reference |  | Reference |  |
| 0.5-1 | 0.866 (0.798, 0.940) | **<0.001** | 0.956 (0.880, 1.039) | 0.289 |
| 2-3 | 0.801 (0.750, 0.855) | **<0.001** | 0.869 (0.813, 0.928) | **<0.001** |
| 4-5 | 0.784 (0.733, 0.838) | **<0.001** | 0.819 (0.765, 0.877) | **<0.001** |
| 6-7 | 0.854 (0.789, 0.923) | **<0.001** | 0.866 (0.799, 0.938) | **<0.001** |
| 8-9 | 0.881 (0.784, 0.991) | **0.035** | 0.851 (0.755, 0.958) | **0.008** |
| ≥10 | 0.962 (0.848, 1.091) | 0.550 | 0.892 (0.784, 1.015) | 0.082 |

Model adjusted for age, sex, ethnicity, education, Townsend deprivation index, body mass index, income, physical activity, sleep duration, smoking status, alcohol intake status, coffee consumption, fish consumption, vegetable consumption, fruit consumption, low-density lipoprotein cholesterol, depression status, hypertension, cardiovascular diseases, diabetes, cancer.

Bold indicates statistical significance (*p*-value < 0.05). The *p*-values are unadjusted for multiple comparisons.

**Supplementary Table 17.** Subgroup analysis of the association between tea consumption and dementia by sex.

| **Tea (cups/day)** | **HR (95% CI)** | ***p* value** |
| --- | --- | --- |
| **Female** |  |  |
| 0 | Reference |  |
| 0.5-1 | 1.035 (0.919, 1.165) | 0.574 |
| 2-3 | 0.891 (0.810, 0.980) | **0.018** |
| 4-5 | 0.865 (0.785, 0.953) | **0.003** |
| 6-7 | 0.972 (0.869, 1.087) | 0.618 |
| 8-9 | 0.886 (0.747, 1.052) | 0.167 |
| ≥10 | 1.020 (0.844, 1.232) | 0.838 |
| **Male** | | |
| 0 | Reference |  |
| 0.5-1 | 0.871 (0.776, 0.977) | **0.018** |
| 2-3 | 0.814 (0.743, 0.893) | **<0.001** |
| 4-5 | 0.748 (0.680, 0.822) | **<0.001** |
| 6-7 | 0.750 (0.669, 0.840) | **<0.001** |
| 8-9 | 0.796 (0.676, 0.938) | **0.006** |
| ≥10 | 0.783 (0.658, 0.931) | **0.006** |
| ***p* for interaction** | **0.023** | |

Model 1: Adjusted for age, sex, and ethnicity.

Model 2: Adjusted for age, sex, ethnicity, education, Townsend deprivation index, body mass index, income, physical activity, sleep duration, smoking status, alcohol intake status, coffee consumption, fish consumption, vegetable consumption, fruit consumption, low-density lipoprotein cholesterol, depression status, hypertension, cardiovascular diseases, diabetes, and cancer.

Bold indicates statistical significance (*p*-value < 0.05). The *p*-values are unadjusted for multiple comparisons.

**Supplementary Table 18.** Subgroup analysis of the association between tea consumption and dementia by age.

| **Tea (cups/day)** | **HR (95% CI)** | ***p* value** |
| --- | --- | --- |
| **Midlife** |  |  |
| 0 | Reference |  |
| 0.5-1 | 1.001 (0.886, 1.130) | 0.993 |
| 2-3 | 0.809 (0.731, 0.894) | **<0.001** |
| 4-5 | 0.803 (0.724, 0.889) | **<0.001** |
| 6-7 | 0.861 (0.763, 0.972) | **0.015** |
| 8-9 | 0.710 (0.588, 0.858) | **<0.001** |
| ≥10 | 0.872 (0.725, 1.049) | 0.147 |
| **Late-life** |  |  |
| 0 | Reference |  |
| 0.5-1 | 0.902 (0.806, 1.010) | 0.074 |
| 2-3 | 0.885 (0.810, 0.966) | **0.006** |
| 4-5 | 0.809 (0.739, 0.886) | **<0.001** |
| 6-7 | 0.852 (0.766, 0.948) | **0.003** |
| 8-9 | 0.945 (0.812, 1.101) | 0.471 |
| ≥10 | 0.884 (0.741, 1.055) | 0.171 |
| ***p* for interaction** | 0.099 | |

Model 1: Adjusted for age, sex, and ethnicity.

Model 2: Adjusted for age, sex, ethnicity, education, Townsend deprivation index, body mass index, income, physical activity, sleep duration, smoking status, alcohol intake status, coffee consumption, fish consumption, vegetable consumption, fruit consumption, low-density lipoprotein cholesterol, depression status, hypertension, cardiovascular diseases, diabetes, and cancer.

Bold indicates statistical significance (*p*-value < 0.05). The *p*-values are unadjusted for multiple comparisons.

**Supplementary Table 19.** Subgroup analysis of the association between tea consumption and dementia by APOE genotype. (n=438,078)

| **Tea (cups/day)** | **HR (95% CI)** | ***p* value** |
| --- | --- | --- |
| **APOE *ε*4 carriers** |  |  |
| 0 | Reference |  |
| 0.5-1 | 0.870 (0.766, 0.988) | **0.031** |
| 2-3 | 0.821 (0.743, 0.908) | **<0.001** |
| 4-5 | 0.803 (0.725, 0.889) | **<0.001** |
| 6-7 | 0.857 (0.760, 0.967) | **0.012** |
| 8-9 | 0.842 (0.704, 1.007) | 0.060 |
| ≥10 | 0.904 (0.741, 1.102) | 0.318 |
| **Non-APOE *ε*4 carriers** |  |  |
| 0 | Reference |  |
| 0.5-1 | 0.993 (0.891, 1.108) | 0.903 |
| 2-3 | 0.876 (0.802, 0.956) | **0.003** |
| 4-5 | 0.797 (0.728, 0.873) | **<0.001** |
| 6-7 | 0.841 (0.756, 0.935) | **0.001** |
| 8-9 | 0.819 (0.699, 0.960) | **0.014** |
| ≥10 | 0.872 (0.738, 1.030) | 0.107 |
| ***p* for interaction** | 0.568 | |

Model adjusted for age, sex, ethnicity, education, Townsend deprivation index, body mass index, income, physical activity, sleep duration, smoking status, alcohol intake status, coffee consumption, fish consumption, vegetable consumption, fruit consumption, low-density lipoprotein cholesterol, depression status, hypertension, cardiovascular diseases, diabetes, and cancer.

Bold indicates statistical significance (*p*-value < 0.05). The *p*-values are unadjusted for multiple comparisons. APOE, apolipoprotein E.

**Supplementary Table 20.** Subgroup analysis of the association between tea consumption and dementia by PRS. (n=404,252)

| **Tea (cups/day)** | **HR (95% CI)** | ***p* value** |
| --- | --- | --- |
| **Low** |  |  |
| 0 | Reference |  |
| 0.5-1 | 0.988 (0.759, 1.286) | 0.928 |
| 2-3 | 0.871 (0.705, 1.077) | 0.203 |
| 4-5 | 0.785 (0.632, 0.976) | **0.029** |
| 6-7 | 0.728 (0.560, 0.947) | **0.018** |
| 8-9 | 0.717 (0.484, 1.063) | 0.098 |
| ≥10 | 0.732 (0.482, 1.112) | 0.144 |
| **Intermediate** |  |  |
| 0 | Reference |  |
| 0.5-1 | 0.905 (0.797, 1.028) | 0.126 |
| 2-3 | 0.861 (0.779, 0.952) | **0.004** |
| 4-5 | 0.790 (0.712, 0.875) | **<0.001** |
| 6-7 | 0.852 (0.756, 0.961) | **0.009** |
| 8-9 | 0.875 (0.734, 1.043) | 0.136 |
| ≥10 | 0.929 (0.772, 1.117) | 0.433 |
| **High** |  |  |
| 0 | Reference |  |
| 0.5-1 | 0.939 (0.823, 1.071) | 0.348 |
| 2-3 | 0.838 (0.754, 0.930) | **0.001** |
| 4-5 | 0.784 (0.704, 0.872) | **<0.001** |
| 6-7 | 0.862 (0.760, 0.976) | **0.020** |
| 8-9 | 0.772 (0.638, 0.934) | **0.008** |
| ≥10 | 0.885 (0.719, 1.089) | 0.247 |
| ***p* for interaction** | 0.926 | |

Model adjusted for age, sex, education, Townsend deprivation index, body mass index, income, physical activity, sleep duration, smoking status, alcohol intake status, coffee consumption, fish consumption, vegetable consumption, fruit consumption, low-density lipoprotein cholesterol, depression status, hypertension, cardiovascular diseases, diabetes, and cancer.

Bold indicates statistical significance (*p*-value < 0.05). The *p*-values are unadjusted for multiple comparisons.

Low (lowest quintile of PRS), Intermediate (PRS quintile 2 to 4), High (highest quintile of PRS). PRS, polygenic risk score.
